# Pemetrexed plus carboplatin in elderly patients with malignant pleural mesothelioma: combined analysis of two phase II trials

**DOI:** 10.1038/sj.bjc.6604442

**Published:** 2008-06-10

**Authors:** G L Ceresoli, B Castagneto, P A Zucali, A Favaretto, M Mencoboni, F Grossi, D Cortinovis, G Del Conte, A Ceribelli, A Bearz, S Salamina, F De Vincenzo, F Cappuzzo, M Marangolo, V Torri, A Santoro

**Affiliations:** 1 Department of Oncology, Istituto Clinico Humanitas IRCCS, Rozzano, Milano, Italy; 2 Department of Medical Oncology, Azienda Ospedaliera, Novi Ligure, Italy; 3 Department of Medical Oncology, Istituto Oncologico Veneto IRCCS, Padova, Italy; 4 Department of Medical Oncology, Ospedale Villa Scassi, Sampierdarena, Italy; 5 Department of Medical Oncology, National Institute for Cancer Research, Genova, Italy; 6 Department of Medical Oncology, Unit 2, National Cancer Institute, Milano, Italy; 7 Department of Medical Oncology, Azienda Ospedaliera, Trieste, Italy; 8 Department of Medical Oncology, Unit A, Regina Elena Cancer Institute, Roma, Italy; 9 Department of Medical Oncology, Unit A, National Cancer Institute, Aviano, Italy; 10 Department of Oncology and Hematology, Istituto Oncologico Romagnolo, Ravenna, Italy; 11 Department of Oncology, Istituto Mario Negri, Milano, Italy

**Keywords:** malignant pleural mesothelioma, elderly patients, chemotherapy, carboplatin, pemetrexed

## Abstract

The incidence of malignant pleural mesothelioma (MPM) in elderly patients is increasing. In this study, pooled data from two phase II trials of pemetrexed and carboplatin (PC) as first-line therapy were retrospectively analysed for comparisons between age groups. Patients received pemetrexed 500 mg m^−2^ and carboplatin AUC 5 mg ml^−1^ min^−1^ intravenously every 21 days with standard vitamin supplementation. Elderly patients were defined as those ⩾70 years old. A total of 178 patients with an ECOG performance status of ⩽2 were included. Median age was 65 years (range 38–79), with 48 patients ⩾70 years (27%). Grade 3–4 haematological toxicity was slightly worse in ⩾70 *vs* <70-year-old patients, with neutropenia observed in 25.0 *vs* 13.8% (*P*=0.11), anaemia in 20.8 *vs* 6.9% (*P*=0.01) and thrombocytopenia in 14.6 *vs* 8.5% (*P*=0.26). Non-haematological toxicity was mild and similar in the two groups. No significant difference was observed in terms of overall disease control (60.4 *vs* 66.9%, *P*=0.47), time to progression (7.2 *vs* 7.5 months, *P*=0.42) and survival (10.7 *vs* 13.9 months, *P*=0.12). Apart from slightly worse haematological toxicity, there was no significant difference in outcome or toxicity between age groups. The PC regimen is effective and well tolerated in selected elderly patients with MPM.

The incidence of malignant pleural mesothelioma (MPM) is increasing in most of the world, and is expected to rise in the next 10–15 years in Europe (Robinson and Lake, 2005). Owing to the long latent period following asbestos exposure, MPM is often diagnosed late in life. A high rate of diagnosis in elderly patients is reported by several mesothelioma registers and epidemiological studies (Price and Ware, 2004; Marinaccio *et al*, 2005). The median age of disease onset in the United States has been recently reported to be 74 years, according to the Surveillance, Epidemiology, and End Results database (SEER Database, 2007). A similar trend has been reported in western European countries (Hodgson *et al*, 2005).

A minority of patients with MPM is eligible for surgical treatment (Sugarbaker *et al*, 1999); most are candidates for chemotherapy during the course of their disease. Recently, the novel multitargeted antifolate pemetrexed was shown to have activity in MPM as a single agent and in combination with platinum compounds (Hughes *et al*, 2002; Scagliotti *et al*, 2003; Vogelzang *et al*, 2003). A large phase III trial testing pemetrexed and cisplatin *vs* cisplatin alone in 448 chemo-naive patients with MPM showed a significant advantage in survival, time to progression (TTP) and response rate (RR) with the combined regimen (Vogelzang *et al*, 2003). Following the results of this trial, the combination of cisplatin and pemetrexed has become established as the standard of care in systemic therapy for MPM (Hazarika *et al*, 2005). However, the typical non-haematological toxicity profile of cisplatin is questionable in the context of a palliative treatment; furthermore, many MPM patients, and especially elderly patients, are unfit to receive cisplatin-based chemotherapy (Zucali and Giaccone, 2006). Therefore, schedules with carboplatin have been explored in an attempt to reduce toxicity, while maintaining the same survival benefit (Hughes *et al*, 2002; Favaretto *et al*, 2003; Ceresoli *et al*, 2006; Castagneto *et al*, 2008). Recently, we published the results of the combination of pemetrexed and carboplatin administered as first-line treatment in 102 patients (Ceresoli *et al*, 2006). In this trial, time to disease progression and overall survival (OS) were similar to the results achieved with the standard regimen of pemetrexed and cisplatin, suggesting that the carboplatin combination could be an alternative option for these patients. These results have been confirmed independently in another trial using the same treatment schedule in 76 chemo-naive patients (Castagneto *et al*, 2008).

Despite the increase in the incidence of MPM with age, elderly patients are under-represented in clinical trials. The median age of the patients enrolled in the cisplatin/pemetrexed phase III trial was 61 years (Vogelzang *et al*, 2003), and the percentage of patients aged ⩾70 years was about 19% (Vogelzang and Symanowski, personal communication). Median patient age was 58 years in another large phase III trial of the combination of cisplatin and raltitrexed *vs* cisplatin alone (Van Meerbeeck *et al*, 2005).

Prospective as well as retrospective data regarding the tolerability and efficacy of anticancer treatments for elderly patients affected by MPM are lacking. The aim of this study was to perform a pooled retrospective analysis using individual patient data from two phase II trials that included patients treated with the same treatment schedule of carboplatin and pemetrexed, comparing the efficacy, toxicity and survival outcomes of this combination in elderly *vs* younger patients. Elderly patients were defined as those ⩾70 years old.

## Patients and methods

### Patient population

The two trials that comprised this pooled analysis were prospective phase II studies conducted between November 2002 and July 2005, which enrolled chemo-naive patients with histologically proven MPM who were not candidates for surgery. Eligibility criteria were nearly identical in the two studies (Table 1), and included an Eastern Cooperative Oncology Group (ECOG) performance status ⩽2 and an estimated life expectancy of ⩾12 weeks. The presence of unidimensionally and/or bidimensionally measurable disease was mandatory. Adequate bone marrow, renal and hepatic function, as assessed by laboratory tests, was required. Prior systemic or intracavitary chemotherapy, documented brain metastases, serious co-morbidities or other malignancies were not allowed. Patients with a measurable recurrence after surgery were considered eligible. Both were age-unspecified trials; the patients had to be aged ⩾18 years. Written informed consent was obtained from each patient. Both studies were conducted after approval by the appropriate ethical review boards.

### Trials and patient selection

**Figure 1:**
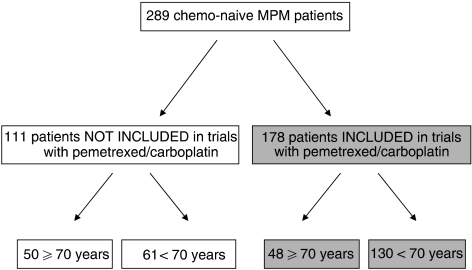
Therapeutic management of the total population of chemo-naive patients with malignant pleural mesothelioma (MPM) referred to the participating centres during the study period. Patients aged ⩾70 years represented 34% (98 of 298) of new cases of MPM.

The patients included in the two trials represented about 60% of the total population of patients with a diagnosis of chemo-naive MPM, referred to the participating centres during the study periods (Figure 1). Patients not enrolled in the two studies were excluded for several reasons, mainly because they were candidates for multimodality programs including surgery, or because they were treated with supportive care only (due to poor performance status) or with other chemotherapy regimens, such as single-agent pemetrexed, cisplatin and pemetrexed or cisplatin and gemcitabine. Overall, nearly half of the patients over 70 years were included in the two trials with pemetrexed/carboplatin. Of the elderly patients not treated with carboplatin and pemetrexed, most received single-agent pemetrexed (17 patients), intrapleural treatments (20 cases) or supportive care only (8 patients).

### Study design

A retrospective analysis was conducted using individual patient data for all of the patients included in the two studies. Follow-up was updated to March 2008. Both studies consisted of a phase II trial of the combination of pemetrexed and carboplatin as first-line treatment in unresectable MPM (Ceresoli *et al*, 2006; Castagneto *et al*, 2008). In study A, patients from eight Italian institutions were enrolled prospectively; study B included patients from three other national centres. According to the original protocols, the primary end point of both studies was tumour RR. Secondary end points included toxicity, time to progressive disease (TTP) and OS. In the pooled analysis, the focus of which was on the effect of age, several different end points were explored. In particular, baseline differences between elderly (⩾70 years) and younger (<70 years) patients were assessed. Moreover, the efficacy, toxicity and survival outcomes of this combination in elderly *vs* younger patients were compared.

### Patient assessment

Baseline assessment for all patients included a complete medical history and physical examination, full blood count and blood chemistry. A CT scan of the chest and abdomen was performed. Patients were staged according to the TNM staging system proposed by the International Mesothelioma Interest Group (Rusch, 1995). Best tumour response was evaluated according to different criteria in the two trials: in trial A hybrid uni/bi-dimensional criteria were used (Vogelzang *et al*, 2003); in trial B the response was assessed according to modified RECIST criteria (Byrne and Nowak, 2004). No confirmatory scans were performed on patients exhibiting partial response (PR) or stable disease (SD) in either study. Overall disease control was defined as the percentage of patients achieving an objective response or SD. Treatment toxicity was evaluated according to the NCI Common Toxicity Criteria (CTC) version 2.0 grading system (National Cancer Institute, 1999). After completion of the study treatment, patients were evaluated with chest and abdominal CT scans every 2 months until disease progression. Patients were also followed up for survival until death or last contact if still alive. No second-line therapy was planned in either trial. Time to progression was defined as time from study entry (first day of study treatment) until time of disease progression (as shown by radiological or clinical examination) or death from any cause. Patients without any evidence of progressive disease were censored at the date of the last follow-up for the purpose of this analysis. Overall survival was calculated as the time from study entry until death from any cause; patients who were alive on the date of last follow-up were censored on that date.

### Treatment

The treatment schedule was the same in the two trials. Pemetrexed was administered intravenously at a dose of 500 mg m^−2^ over 10 min, followed by carboplatin, administered by a 30-min intravenous infusion at an AUC of 5 mg ml^−1^ min^−1^. Both drugs were given on day 1, every 21 days. All patients received standard vitamin supplementation with folic acid and vitamin B_12_ plus steroid prophylaxis. Standard antiemetic therapy with intravenous 5-HT_3_ antagonists was given before chemotherapy.

### Statistical analysis

Differences in baseline characteristics, rates of response and adverse events in the two groups (⩾70 *vs* <70-year patients) were assessed with Fisher's exact test (Agresti, 1992). Ninety-five per cent confidence intervals for RRs were calculated (Leemis and Trivedi, 1996). Actuarial survival curves were generated using the method of Kaplan and Meier (Kaplan and Meier, 1958). Time to progression and OS in the ⩾70 and <70-year groups were compared through the Mantel–Cox version of the log-rank test (Cox, 1972). Multivariate analysis using a proportional hazard model was also used to estimate the effect of age on survival after controlling for related covariates. All probability values were two-sided. Statistical analysis was performed using the software package SAS, version 9.1.3.

## Results

### Patient characteristics

A total of 178 patients were included in the analysis. The median age of the study population was 65 years (range 38–79). Forty-eight patients (27% of the whole study population) were ⩾70 years old; 11 patients (6%) were ⩾75 years old. Patient characteristics in terms of gender, ECOG performance status, histology and stage were similar in the two age groups (Table 2). Most patients had a performance status ⩽1 and epithelial histological subtype.

### Efficacy

All 178 patients were assessable for best tumour response, which was established according to an intent-to-treat analysis. No patient experienced a complete response in the ⩾70 years group, whereas a PR was achieved in seven patients, for an objective RR of 14.6% (95% CI:6.1–27.8%). In the <70 years group, five patients had a complete response and 26 a PR; the RR was 23.8% (95% CI:16.8–32.1%). The RRs did not differ significantly between the two age groups (*P*=0.15). A similar proportion of patients had SD in the two cohorts: 22 in the ⩾70 years group (45.8, 95% CI:31.4–60.8%) and 56 in the <70 years group (43.1, 95% CI:34.4–52.0%) (*P*=0.86). Overall disease control was also not significantly different: 60.4% of the elderly patients (95% CI:45.3–74.2%) achieved disease control *vs* 66.9% (95% CI:!58.1–74.9%) of their younger counterparts (*P*=0.47).

**Figure 2:**
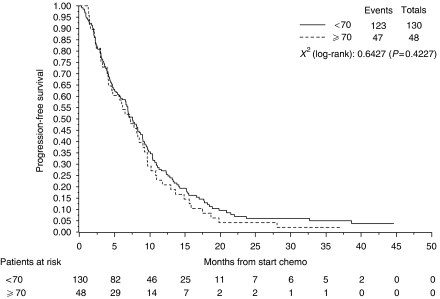
Kaplan–Meier curve of time to disease progression for younger (solid line) and elderly (dotted line) patients (*P*=0.42).

**Figure 3:**
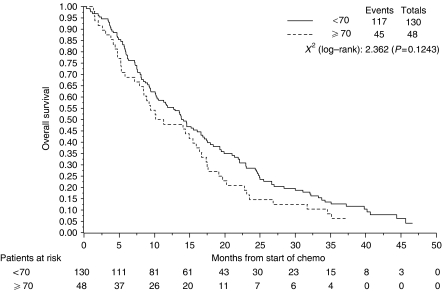
Kaplan–Meier curve of overall survival time for younger (solid line) and elderly (dotted line) patients (*P*=0.12).

With a median follow-up of 37.1 months, 16 patients were still alive, 8 of whom were without any evidence of disease progression. The median TTP and OS for the whole study population were 7.4 and 13.8 months, respectively. Time to progression and OS did not differ significantly between the two age cohorts even after adjusting for different prognostic factors, such as gender, PS, stage and histology. Figure 2 shows the actuarial TTP curves for the two age groups; the median TTP for patients ⩾70 and <70 years were 7.2 and 7.5 months, respectively (*P*=0.42). Younger patients had longer median OS (13.9 months) than those aged ⩾70 years (10.7 months), but the difference was not statistically significant (*P*=0.12) (Figure 3). The 6-months and 1-year estimates of survival were 69 and 48% for patients ⩾70 years, and 76 and 55% for those aged <70 years.

### Toxicity

Patients aged ⩾70 and <70 years received a median of six cycles of treatment; the ranges were 1–12 cycles for elderly and 1–13 cycles for younger patients, respectively. Forty elderly patients (83%) completed at least four cycles, as compared to 109 (84%) of younger patients. Haematological toxicity by age group is summarized in Table 3. Grade 3–4 neutropenia was slightly worse in elderly patients (25.0 *vs* 13.8%; *P*=0.11); however, febrile neutropenia was observed less frequently in this age group (2.1 *vs* 3.8%). Severe anaemia was significantly more frequent in older patients (20.8 *vs* 6.9%; *P*=0.01), occurring mainly as cumulative toxicity. Grade 3–4 thrombocytopenia occurred in 14.6% of the elderly *vs* 8.5% of the younger patients (*P*=0.26). When all toxicity grades were considered (data not shown), neutropenia and anaemia rates remained higher in the elderly patients (*P*=0.03 and *P*=0.0009, respectively), while thrombocytopenia did not differ significantly between the two groups (*P*=0.73). Non-haematological toxicity was mild and similar in the two groups (Table 4). Nausea and vomiting, fatigue, conjunctivitis and diarrhoea were the most commonly reported adverse effects of treatment (Table 4). One case of acute rhabdomyolysis with slow resolution was observed in a 68-year-old male patient (Ceresoli *et al*, 2006; Ceribelli *et al*, 2006). No treatment-related deaths were observed in either of the age groups.

## Discussion

Elderly patients account for the majority of all new cancer cases. According to an estimate, 61% of new cancer diagnoses and 70% of all cancer deaths occur in people aged 65 years or older (Hutchins *et al*, 1999). In the US and in Europe, the number of cancer cases in older people is expected to increase, as they constitute the most rapidly growing section of the population (Yancik and Ries, 2000). However, there is substantial under-representation of these populations in clinical trials, particularly as regards patients aged ⩾70 years (Hutchins *et al*, 1999; Lewis *et al*, 2003; Talarico *et al*, 2004; Kumar *et al*, 2007). This leads to uncertainty about the applicability of the results of studies to elderly patients in terms of both the efficacy and toxicity of treatments. In MPM, older age has been generally considered a negative prognostic factor (Herndon *et al*, 1998; Gatta *et al*, 2006). Apart from age-related biological differences, this may also have been influenced by a nihilistic attitude to treatment of this disease (Treasure and Sedrakyan, 2004). As a result, specific trials for elderly patients affected by MPM are completely lacking, and few data are available to the clinician to assist in making rational treatment decisions.

Our retrospective analysis showed that patients aged ⩾70 years can derive as much benefit from chemotherapy with pemetrexed plus carboplatin as younger patients, with an acceptable burden of toxicity. Pemetrexed and carboplatin are characterized by a favourable toxicity profile in older patients (Weiss *et al*, 2006; Lichtman *et al*, 2007), although changes in renal function with age must be taken into account before their administration. In MPM, the combination is a valuable alternative to the standard regimen of pemetrexed and cisplatin, with similar survival benefit (Hughes *et al*, 2002; Ceresoli *et al*, 2006; Castagneto *et al*, 2008). In a recent report on more than 1700 chemo-naive MPM patients treated within the International Expanded Access Program, a nonrandomized open-label safety study of pemetrexed as a single agent or in combination with platinum derivatives, the combination of pemetrexed with either carboplatin or cisplatin resulted in similar efficacy and tolerability (Santoro *et al*, 2007).

In our series, the baseline patient and disease characteristics were similar between the two age groups; most patients had good performance status, while data on co-morbidity were not available, raising the possibility of a selection bias to include only fit elderly patients (Extermann and Hurria, 2007). Notably, only 11 patients were ⩾75 years; therefore, the results of our study should be regarded with caution with respect to the ‘oldest of the old’, because no consistent data are available on the risk/benefit ratio of pemetrexed and carboplatin chemotherapy in this small subset of patients.

The radiological regression and overall disease control rates did not differ significantly between patients ⩾70 and <70 years, although different response criteria were used in the two trials and response classification was not reviewed centrally. However, in view of the difficulties in assessing radiological response to therapy, the survival outcomes seem the best treatment end points in MPM (Francart *et al*, 2006; Ceresoli *et al*, 2007a). The median TTP was similar in the two age groups (7.2 and 7.5 months for patients ⩾70 and <70 years, respectively), with curves nearly overlapping (Figure 2). Younger patients lived longer than elderly (13.9 *vs* 10.7 months), but the difference was not statistically significant. No second-line therapy was planned in the two trials, and this may have influenced OS data. However, the survival benefit of second-line therapy in MPM is not proven (Ceresoli *et al*, 2007b).

Therapy with carboplatin and pemetrexed was well tolerated in both age groups (Tables 3 and 4), with similar dose delivery. Non-haematological toxicity was negligible and did not differ significantly between groups. The haematological toxicity was slightly worse in the elderly population, with a higher rate of grade 3–4 neutropenia and anaemia (Table 3). However febrile neutropenia, which is commonly used as surrogate for unacceptable toxicity, was uncommon and was not increased in the elderly group. Moreover, no toxic death was reported.

In conclusion, our data suggest that chemotherapy with pemetrexed and carboplatin is effective and safe in elderly patients with good performance status affected by MPM. Extra caution is required for patients aged ⩾75 years due to the lack of data in this small age subgroup. Prospective evaluation of this regimen in specific trials on elderly MPM patients is warranted.

## Figures and Tables

**Table 1:** Main trial characteristics

	**Trial A**	**Trial B**
	**Ceresoli *et al* (2006)**	**Castagneto *et al* (2008)**
Accrual period	November 2002– March 2005	July 2003–July 2005
Total accrual, no. of patients	102	76
No. of patients ⩾70 years	32	16
Age restrictions	⩾18 years	⩾18 years

**Table 2:** Baseline patient characteristics by age group

	**Patients ⩾70 years (*N*=48) (%)**	**Patients <70 years (*N*=130) (%)**	***P***
*Gender*			
Male	33 (69)	98 (75)	0.37
Female	15 (31)	32 (25)	
			
*ECOG performance status*			
0	16 (33)	52 (40)	0.43
1	28 (58)	69 (53)	
2	4 (8)	9 (7)	
			
*Histology*			
Epithelial	36 (75)	101 (78)	0.70^*^
Mixed	4 (8)	17 (13)	
Sarcomatoid	5 (10)	5 (4)	
Unclassified	3 (6)	7 (5)	
			
*IMIG stage*			
Stage I	2 (4)	5 (4)	0.34
Stage II	7 (15)	10 (8)	
Stage III	16 (33)	45 (34)	
Stage IV/relapse after EPP	23 (48)	70 (54)	

^*^Epithelial *vs* non-epithelial patients.

**Table 3:** Grade 3–4 haematological toxicity by patient

	**Patients ⩾70 years (*N*=48)**				**Patients <70 years (*N*=130)**				
	**G3 (No.)**		**G4 (No.)**	**G3–4 (%)**	**G3 (No.)**		**G4 (No.)**	**G3–4 (%)**	***P***
Neutropenia	5		7	25.0	13		5	13.8	0.11
Anaemia	9		1	20.8	7		2	6.9	0.01
Thrombocytopenia	6		1	14.6	8		3	8.5	0.26
Febrile neutropenia		1		2.1		5		3.8	

**Table 4:** Non-haematological toxicity by patient

	**Patients >70 years (*N*=48)**			**Patients <70 years (*N*=130)**		
	**G1 (No.)**	**G2 (No.)**	**G3 (No.) (%)**	**G1 (No.)**	**G2 (No.)**	**G3 (No.) (%)**
Nausea/vomiting	18	6	2 (4.1)	37	24	8 (6.2)
Fatigue	10	9	1 (2.1)	26	13	4 (3.1)
Diarrhoea	1	0	1 (2.1)	2	1	4 (3.1)
Conjunctivitis	5	2	1 (2.1)	15	3	1 (0.8)
Stomatitis	5	4	0	11	5	1 (0.8)
Fever	2	1	1 (2.1)	5	3	3 (2.3)
